# 3D surface morphology imaging of opaque microstructures via light-field microscopy

**DOI:** 10.1038/s41598-018-28945-2

**Published:** 2018-07-12

**Authors:** Yong Da Sie, Chun-Yu Lin, Shean-Jen Chen

**Affiliations:** 1 Department of Engineering Science, National Cheng Kung University, Tainan, 701 Taiwan; 2 Advanced Optoelectronic Technology Center, National Cheng Kung University, Tainan, 701 Taiwan; 3 College of Photonics, National Chiao Tung University, Tainan, 711 Taiwan

## Abstract

Observing dynamic micro-scale phenomena occurring at millisecond time scales, such as organism activity, micron particle flows, or any opaque object observation, requires volumetric microscopy techniques able to achieve high data acquisition rates while maintaining contrast so that measurement of fine micro-scale features is possible. In realizing this purpose, the light-field (LF) technique has already been used on three-dimensional (3D) scene capturing and even for microscopic visualizations. In studying the ability and feasibility of 3D surface morphology reconstruction via LF microscopy, we adopted a lab-made LF microscope and integrated a four-dimensional Fourier slice algorithm and a Markov random field propagation algorithm. Furthermore, for numerical comparison and quantized analysis, the Tenengrad function was utilized to calculate the average contrast of the region of interest. Reflective US Air Force targets and 3D photolithography-made micro-scaffolds coated with 50 nm nickel thin films were adopted for system alignment and calibration. The experimental results demonstrate that the developed LF microscope with the signal processing algorithms can observe the 3D surface morphology of opaque microstructures with one snapshot, and has been preliminary applied to Brownian motion observation with 30 Hz volumetric image rate.

## Introduction

Three-dimensional (3D) micro-surface morphology measurement is currently used in many fields, such as dynamics of micro-electro-mechanical systems, biomedical applications for label-free live cells, and detection of hydrodynamic flow of micron-size particles, among others^1–7^. In the case of microelectromechanical systems (MEMS) 3D surface measurement, the difference between vertical laser scanning interferometry and digital holographic microscopy (DHM) is discussed. For dynamic observation considerations, DHM has greater potential for wide-field image observation, and can increase the data recording rate. Techniques for such measurement include DHM, laser scanning microscopy for 3D topography, and quantitative phase microscopy for transparent samples. Unlike fluorescence laser scanning microscopy, these techniques are usually applied to unstained or label-free specimens. Moreover, DHM allows a full 3D registration with wide-field exposure to be obtained, but without any of the vertical displacement that occurs, for instance, in vertical laser scanning interferometry, according to white light sources and phase shifting interferometry^1^. However, these techniques involve complex optical setups with two-beam interference optical designs, where one beam is the object beam and the other is the reference beam, which necessitates a very coherent light source. To obtain the 3D information in DHM, phase reconstruction and detection must be taken; furthermore, many phase-shift images need to be recorded to calculate the phase information. In doing so, however, mass image detection tends to increase the recording time and decrease the data acquisition rate in practical application. To address these challenges, the plenoptic, or light-field (LF) technique, might be one solution to this issue, and does not need a coherent light source.

LF, also known as the plenoptic technique, was developed in the 1990s for camera application^8–11^. For microscopy application, development has occurred over thedecade^12,13^. The main idea of LF is to instantaneously record all information of light travelling in a 3D space^8–14^. To describe light travelling in a 3D space via a two-dimensional detector, four-dimensional (4D) coordinates can be used, including two domains for the angular space and two domains for the horizontal space. Through the 4D coordinate system, the 3D image of the object can be digitally refocused via the light-field 4D Fourier slice theorem with a heterodyned or microlens array (MLA) light-field configuration^15,16^. In the digitally-refocused LF configuration, an alpha coefficient can be defined as the relative depth of the sensor plane. More specifically, the relative depth is the distance between the image lens and the digitally-refocused virtual image; meanwhile, the ratio between this distance and the length from the sensor plane to the image-lens plane is defined as the alpha coefficient. To build a light-field microscope with an infinity imaging system, the *f*-number of each microlens should fit the numerical aperture of the objective, thereby optimizing the spatial angle. To calculate 3D-section images from conventional microscopy, the approach to maximum-likelihood (ML) image deconvolution is equivalent to minimizing the generalized Kullback-Leibler (KL) divergence, for which an iterative algorithm converging to nonnegative minimizers of the KL divergence is the well-known Richardson-Lucy (RL) algorithm^17–19^. In conventional optical microscopy, a blurred image can be considered as the convolution of the optical system impulse response (or called the intensity point spread function (IPSF) in incoherent image formation) and object. However, in LF microscopy, the relation between an object and image is more complex; furthermore, the aliasing problem also exists due to down-sampling the MLA. To solve these above problems, the 3D LF-deconvolution method can be applied to reconstruct 3D images from 4D LF images; however, in doing so, a five-dimensional (5D) measurement matrix modelling requires predicting to describe the relations between a 3D object and two-dimensional (2D) LF image, which in turn requires the use of oversampling and resampler filters to inhibit the aliasing problem^20–23^. With the support of measurement-matrix modelling, the iterative RL algorithm can be employed to reconstruct 3D-section images from the 2D LF images, although the relation between the object and image is not convoluted.

3D LF deconvolution can readily adapt to reconstruct 3D fluorescence images. Nevertheless, this approach, exploiting the above mentioned method, is suitable for incoherent fluorescence microscopy, where the volume to be reconstructed is largely transparent (i.e. with minimal scattering or absorption)^19,24^. For the case of opaque objects or partial coherence under a low-numerical aperture objective condition, a digital refocused algorithm, such as the 4D Fourier slice theorem, can still function well. Therefore, this situation can be seen as a computer vision issue. In solving 3D morphology problem, the Markov random field (MRF) theorem has been widely used in computer vision. Based on the Markov property, the probability of the whole image-organization space can be simplified and described via Gibbs distribution. To obtain the ML or MAP probability requires acquiring the arguments of the minima energy function^25^. To calculate the 3D surface morphology from the 4D LF refocused image, a method that combines defocus and the correspondence LF image can be used to calculate the distribution of the 3D image^26,27^; furthermore, the relation between the alpha coefficient of the Fourier slice theorem and the real distance can be calibrated via the image-quality versus alpha curve^28,29^.

In this research, we demonstrate that calculation of the 3D surface geometry of an opaque can be approached via refocused images of LF microscopy, after a computer vision method is used to estimate depth image. Finally, the depth image is adapted into a real-distance image via calibration of the alpha coefficient. To evaluate a depth image from LF microscopy, the 4D Fourier slice algorithm is used to obtain a series of digitally refocused images that allow the defocused and correspondence image to be calculated from those images. The MRF propagation method is then used to acquire the 3D depth image^26^. Thereafter, the image-quality versus alpha curve is employed to perform calibration via a USAF target shifted to different distances, as described below. In the system calibration, the micro-3D opaque objects are made by multiphoton fabrication of Norland Optical Adhesive 81 (NOA-81) on glass slides and coated with a 50 nm Ni thin film^30^. Accordingly, in this work, we investigate and demonstrate the feasibility of utilizing LF microscopy to reconstruct the 3D micro-morphology of opaque objects and observe the 3D Brownian motion of polystyrene microspheres.

## Results

### LF System Characterization and Alignment

#### USAF target of focusing range calibration

To examine the refocusing depth region and calibrate the practical LF-microscopy distance, USAF targets were employed at different focus positions with reflective lighting. The 4D Fourier slice algorithm was utilized here to obtain a series of different digitally refocused images. Measurement of the precise stage-axial movement was achieved via a mechanical micro-displacement meter.

**Figure 1:**
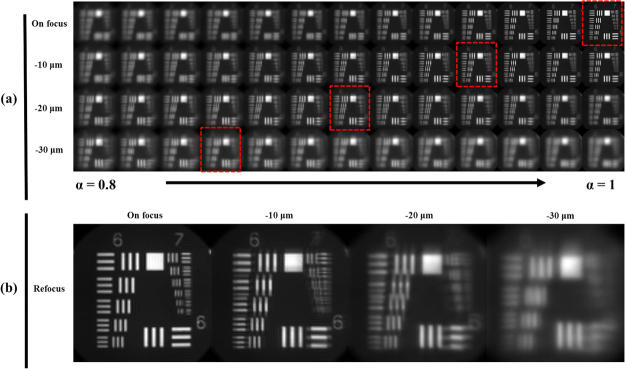
USAF target experiment for examine refocusing depth region. (**a**) Digital refocused image of different practical focused position separately are on focus, −10 μm, −20 μm, and −30 μm. The horizontal coordinate shows different alpha coefficient value from 0.8 to 1, which means from lower position to on focus. (**b**) Exact digital refocused image of different practical focused image, which are also shown in the red dot block of (**a**).

**Figure 2:**
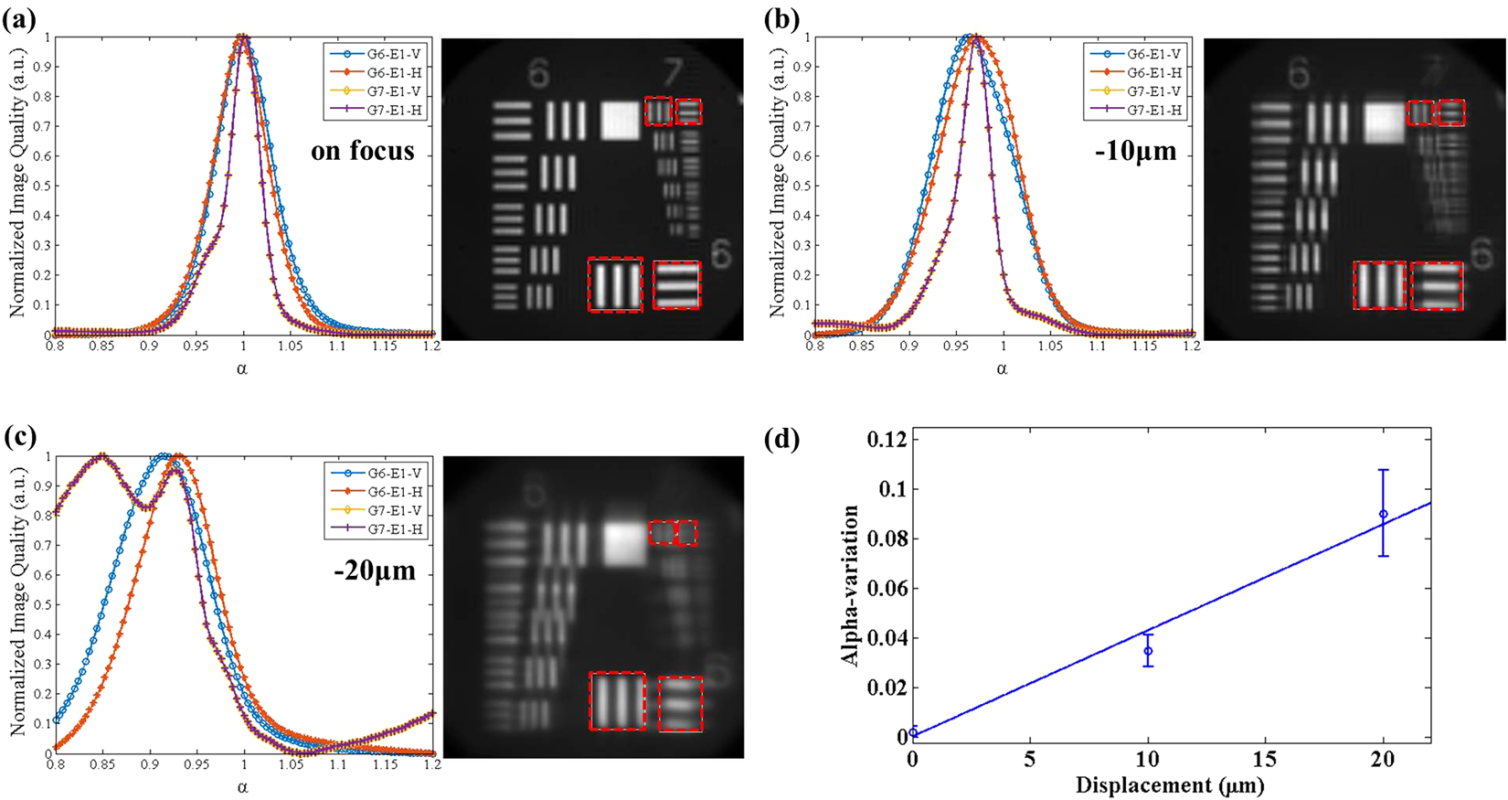
Utilize USAF target to calibrate practical distance of LF microscopy, (**a**) when USAF target is placed on the focusing, (**b**) −10 μm related toon focus, (**c**) −20 μm related toon focus. Each curve is analysed with Tenengrad function, and select different ROI to process, which are group 6 element 1 vertical (G6-E1-V) (blue curve), group 6 element 1 horizontal (G6-E1-H) (red curve), group 7 element 1 horizontal (G7-E1-H) (yellow curve), and group 7 element 1 horizontal (G7-E1-H) (purple curve) as shown in the red dot block area. (**d**) Plot of alpha-variation versus depth displacement. According to the results from parts (**a**–**c**), the best image quality of each ROI is chosen to plot the alpha-variation versus displacement. Linear curve fitting is used to fit the averaged alpha-variation data at different displacements related to the focus position.

Figure 1 shows the results of the different digital-refocused images of different real-focusing locations taken from the focused position to 30 μm lower than the focused position. Due to the operation axial region of the experiment being only on one side of the focus position, the coefficient alpha of the digitally-refocused image was chosen from 0.8 to 1, which means from the lower-focus position to the focused location. Figure 1(b) shows different exact digitally-refocused images of various real axial positions, which are highlighted with red-dashed squares in Fig. 1(a). For example, in Fig. 1(b), the on-focus image corresponds to the red-dashed image of the first raw in Fig. 1(a); similarly, the −10 μm image means the exact refocused image at 10 μm lower than the focused position. To avoid subjective judgement of the focused image, definition of these exact refocused images was obtained according to an analysis similar to that in Fig. 2, with the image quality evolution of the Tenengrad function based on the image quality analysis of element 1 from group 6 of the USAF target. From the results of Fig. 1, the clear refocused image will shift due to displacement of the image along the axial direction, the shift value of which is proportional to the practical position. In this manner, it can be observed that the change in alpha value is proportional with the constant displacement which of 10 μm. However, from observing the different groups and elements of refocused USAF targets, the resolution of a refocused image becomes worse than an image which is out of focus.

From the results of Fig. 2, the image quality is defined with the Tenengrad-function curve, which is analysed with each region of interest (ROI) of the right side. Although the out of focus image can be digitally refocused, Fig. 1(b) shows that the image quality is still unclear, which is the cause of the variation change for each curve in Fig. 2. Furthermore, this might also produce some pseudo-signals in judgement when the blurring is too great, which caused the double peak of G7 in Fig. 2(c). Each curve of different ROI after normalization has one maximum value representing the best image quality and revealing the exact focus position. From these curves, calibration of the real distance can be performed. In this research, the average maximum value for each of the four ROI curves was applied to calibrate and acquire the real depth image. The results in Fig. 2(a) indicate that all of the ROIs have the best image quality when alpha equals 1, which means the USAF target is on focus. In Fig. 2(b), when the USAF target is −10 μm from the focused position, the averaged-maximum value of the image quality versus alpha curve shifts to around 0.966; moreover, as shown in Fig. 2(c) the alpha value shifts to around 0.91 at −20 μm away from the focused position. From the measurements and equation (1), the ratio between the displacement (∆*p*) and variation of alpha (∆*α*) can be obtained from the reciprocal of the slope according to the linear curve fitting in Fig. 2(d), the value of which is around 233.88 (μm per *α*). According to Fig. 2, in practice, the ratio of the alpha-value variation and real displacement is linear. With this calibration, the practical 3D morphology can be automatically calculated from the LF microscope image via digital refocusing and combining the defocus and correspondence image for MRF evaluation.

#### 3D micro-scaffold verification for low contrast bright filed image

**Figure 3:**
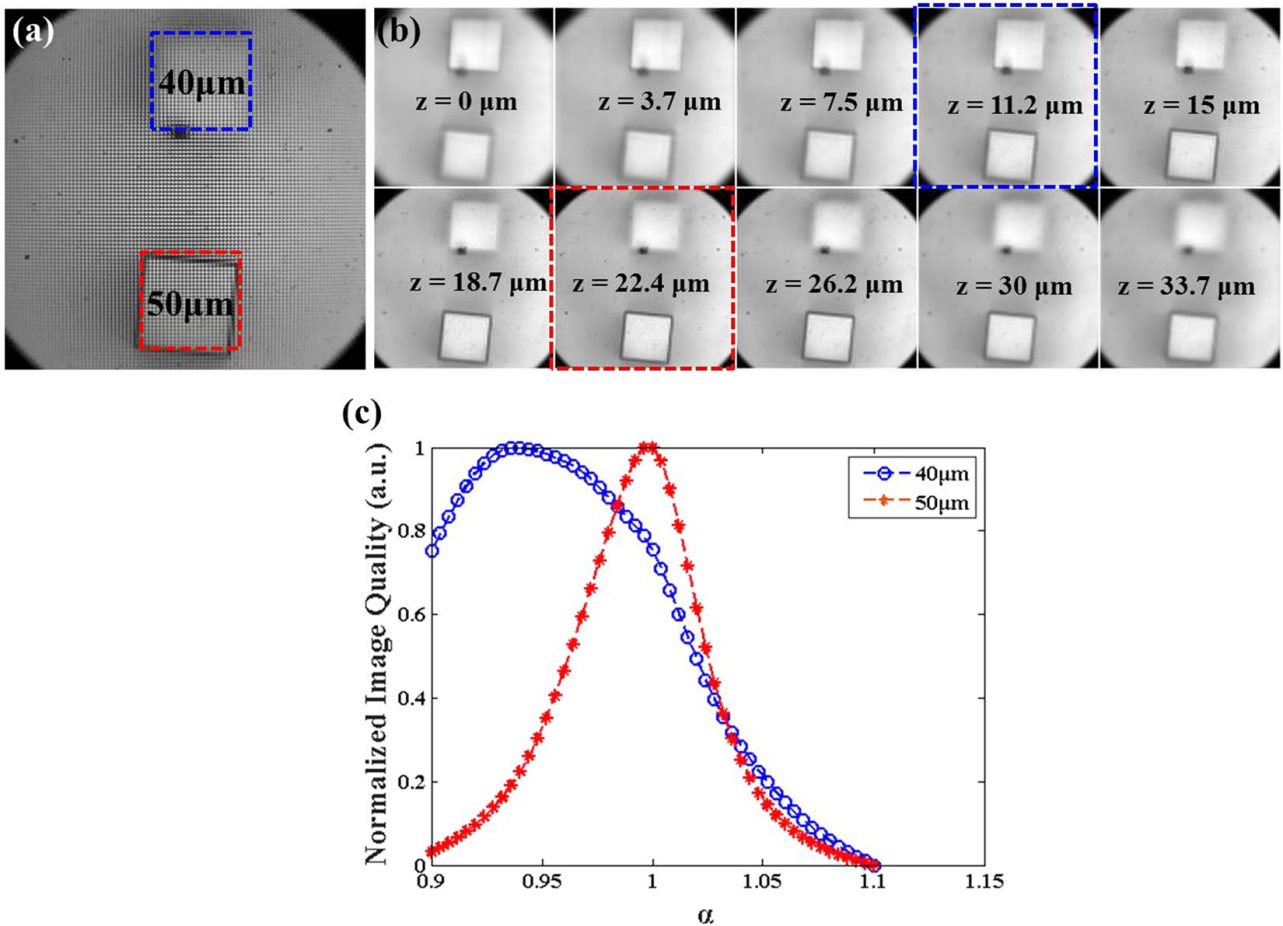
3D micro-scaffold difference of height measurement for low contrast bright filed image. (**a**) CMOS sensor image of two 3D cubic, the area is 75 × 75 μm^2^, and height are 40 μm (blocked by blue dot square) and 50 μm (blocked by red dot square) respectively. (**b**) Digital refocused image of part (**a**) at different depth from 0μm to 33.7 μm. (**c**) Image quality versus alpha value curve analysed from two ROI (blue and red dot block of part (**a**)), from the results of part (**c**), two best digital refocused images are marked in part (**b**) with blue and red dot blocks (correspond to the color of curves).

After the distance calibration, another 3D micro structure, made by NOA-81 and coated with a 50 nm Ni thin film, was employed to examine the relation between the alpha value and real distance under the low-contrast condition. From the CMOS sensor image of Fig. 3(a), there are two cubes, the areas of which are 75 × 75 μm^2^ while the heights are 40 (blue) and 50 μm (red). The purpose of this experiment is to recreate the real reflective microscope conditions, which are unlike the USAF targets, because areal opaque object scatters light in all directions and abundant a lot of background signals in the image, thereby resulting in low-contrast LF images. Therefore, a highly reflective sample was used to simulate low-contrast images, while applying the same image quality analysis as before to identify the best focal position of the digitally refocused image, after which the change in alpha value was compared with the different heights of the two cubes.

As shown in Fig. 3, the height difference between these two cubes was around 10 μm. More specifically, the analyses in Fig. 3(b,c) indicate the maximum image quality of alpha values for the optimized image quality of the 40 and 50 μm height cubes were 0.948 and 0.996, respectively. Accordingly, the value difference of these two alphas is close to 0.048 and the equivalent depth is around 11.2 μm, which approaches the calibration results of the previous experiment. It should be noted that the small error could be attributed to variances in manufacturing or measurement. Furthermore, in Fig. 3(c), the blue curve is smoother than the red curve. This phenomenon is identical to that in Fig. 1, and is because the digital-refocus ability decreases along the axial direction. From these results, we know that the bandwidth cutoff frequency of the LF microscope is decreased along the defocused position. This phenomenon is also discussed in 3D-deconvoluiton LF microscopy^20^ as a band-limit issue.

In this section, the calibration of the LF optical image system is completed via two experiments, one is for the high-contrast condition with the USAF targets, and the other is for the low-contrast case, executed via highly-reflective 3D micro-scaffolds to mimic the conditions of an opaque object. The results of these two experiments were very consistent. As such, based on the analysis in this section, the next step is to evaluate the 3D morphology of an opaque object.

### 3D Surface Morphology Reconstruction

#### Micro 3D morphology reconstruction of cubies

In this section, two cases of different 3D micro-scaffold designs, which were coated with Ni thin film to increase their reflectivity, are used to demonstrate the 3D morphology reconstruction ability of the proposed technique. In these two cases, the 3D morphology is reconstructed and utilizes pseudo-color to illustrate the height via a micro-meter scale.

**Figure 4:**
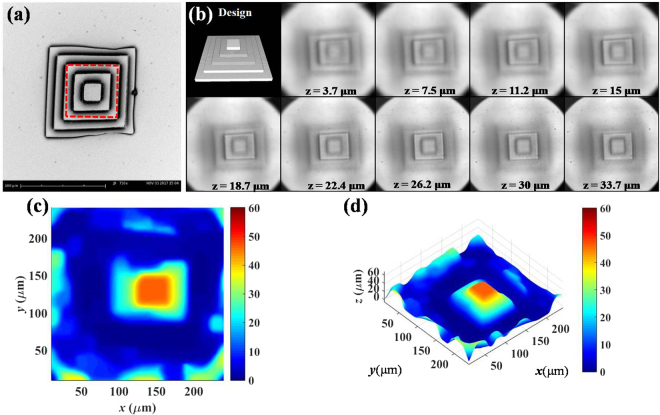
Step pyramid 3D morphology reconstruction experiment. (**a**) SEM image. Red-marked area indicates the 3D reconstructed region. (**b**) Top left is the 3D computer add design (CAD) image. The others are different digital refocused image at different depth from 3.7 μm to 33.7 μm. (**c**) Top view of 3D reconstructed morphology. (**d**) Isometric projection view of 3D reconstructed morphology. Rainbow pseudo color is used to illustrate different depth of object with micro-meter scale.

Figure 4(a) shows the scanning electron microscope (SEM) image of the developed microstructure. The top-left of Fig. 4(b) is a scaffold designed as a step pyramid, in which the height change of each layer is around 10 μm. There are a total of five layers, and from bottom to top, the areas are 200 × 200 μm^2^, 160 × 160 μm^2^, 120 × 120 μm^2^, 80 × 80 μm^2^, and 40 × 40 μm^2^ respectively. This experiment focuses on the top square, and takes an LF-image snapshot. The overall refocusing alpha range for the 3D-image reconstruction was from 0.8 to 1.05, with alpha steps of 0.002; however, for demonstration purposes, the depth range of the digitally refocused images shown in Fig. 4(b) is from 3.7 μm to 33.7 μm. From the results of Fig. 4(b), the different focusing depth images for the top two layers can be clearly seen, i.e. the digitally-refocused images in Fig. 4(b) can represent the 2D morphology of the red-marked area in Fig. 4(a). Figure 4(c,d) present different points of view of the 3D reconstructed image from Fig. 4(b), which again represents the red-marked area of Fig. 4(a). Although the 3D morphology can be reconstructed, only the edges can be approximately reproduced. Therefore, the digitally-refocused images in Fig. 4(b) accurately represent the microstructures with a lateral resolution better than 2 μm. Nevertheless, a few irregularities remain in the 3D reconstructed images in Fig. 4(c,d), which could be reduced by developing a sophisticated algorithm with deep learning.

In this case, the scaffolds are stacked together. Furthermore, due to the contrast of the center area being difficult to distinguish, there remain some artefacts such as ghosting and too much smoothness in some areas; nevertheless, the top two layers of the 3D structure can still be identified. In this case, the proper MRF parameters are critical for the trade-off between ghosting and over-smoothing. Although increasing the smoothness and flatness parameters can reduce the ghosting, doing so also induces some over-smoothing issues. Also, the accuracy of the estimated height is observed to be worse when the values of the flat and smoothness parameters are increased. To investigate in greater detail, a series of un-stacked and separate scaffolds are designed in the next experiment. In addition, some artefacts at the borders of Fig. 4(c,d) appear due to the addition effect of the windowing method of the depth cues and further amplification via the over-smoothing process.

#### Micro 3D morphology reconstruction of various tubes

**Figure 5:**
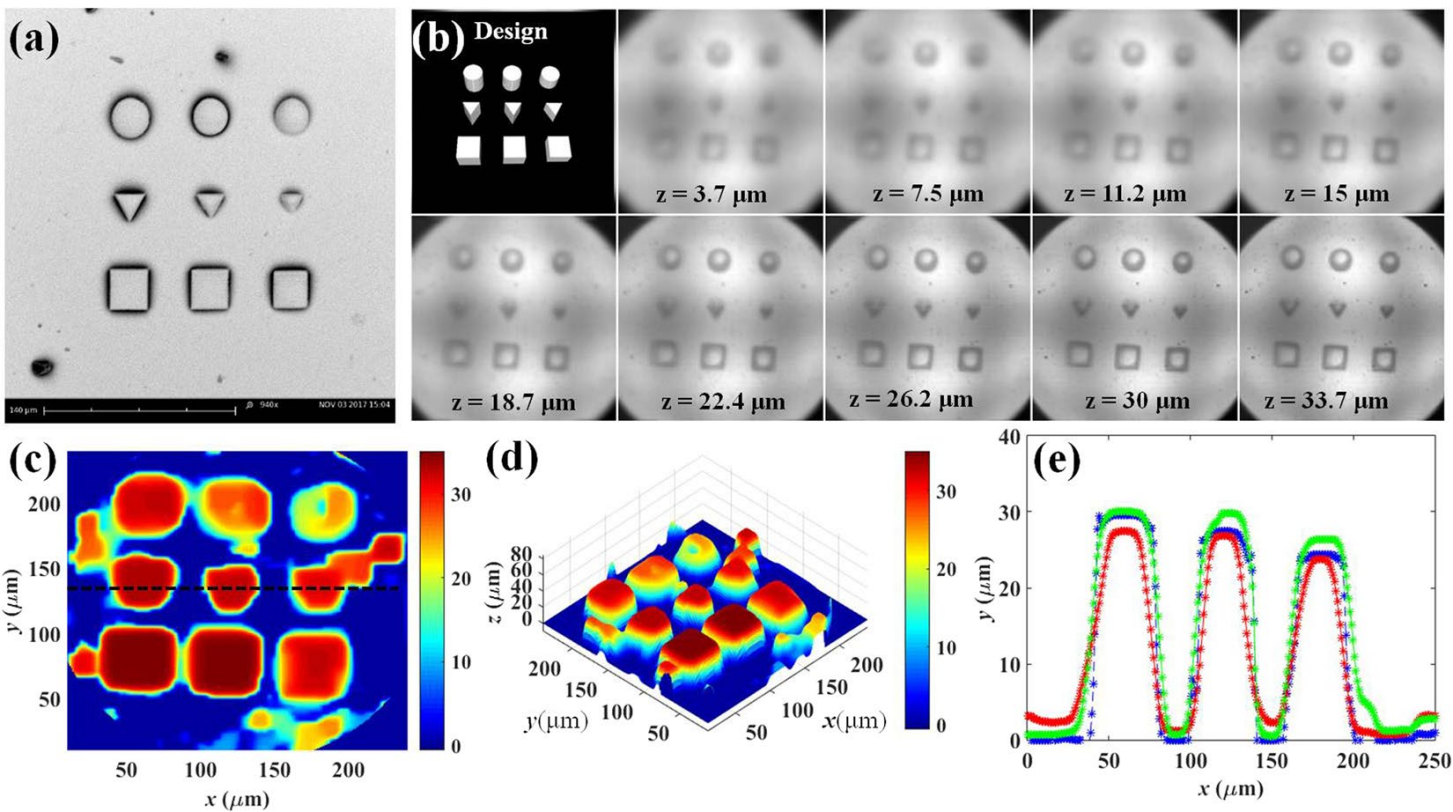
A series of separately circle, triangle, and square column object 3D morphology reconstruction experiment. (**a**) SEM image. (**b**) Top left is the 3D CAD image of these scaffolds. The others are different digital refocused image at different depth from 3.7 μm to 33.7 μm. (**c**) Top view of 3D reconstructed morphology. (**d**) Isometric projection view of 3D reconstructed morphology. Rainbow pseudo color is used to illustrate different depth of object with micro-meter scale. (**e**) The profile of the black-dashed line of part (**c**) and a comparison with different flat and smoothness parameters (*λ*_flat_ = *λ*_smooth_ = 1.1: green-dashed line, *λ*_flat_ = *λ*_smooth_ = 1.2: blue-dashed line, and *λ*_flat_ = *λ*_smooth_ = 1.5: red-dashed line).

The second case is shown in Fig. 5(a), the SEM image of the developed microstructures. The top-left of Fig. 5(b), in which scaffolds were designed as a series of un-stacked and separate columns, comprises circles, triangles, and squares, from top to bottom, respectively. In addition, from left to right, the columns have different heights, namely 30 μm, 27.5 μm, and 25 μm respectively. The overall digital refocusing alphas for the 3D image reconstruction range from 0.85 to 1, with alpha steps of 0.002. As before, the depth of the digitally refocused images showed in Fig. 5(b) range is from 3.7 μm to 33.7 μm for demonstration purposes.

From the results in Fig. 5(b), the different focused images of the different micro-structures can be clearly seen. Figure 5(c,d) present different points of view for the 3D reconstructed image. The 3D surface morphology was successfully reconstructed for each scaffold column. From Fig. 5(c,d), the circles and squares in their respective columns can be easily identified; however, the sharpness of the triangles is slightly less. Figure 5(e) shows the profile of the black-dotted line in Fig. 5(c), in which the change in observation height ranges from 30 μm down to 25 μm, with the height step designed to be around 2.5 μm. Furthermore, different values of the flat and smoothness parameters are utilized with 1.1, 1.2, and 1.5 to examine the trade-off between the accuracy of the estimated height and the reduction of artefacts. By comparing the green and red lines in Fig. 5(e), it can be seen that the over-smoothing process results in fewer artefacts on the right side, but less accuracy of the estimated height overall. Herein, the blue line with 1.2 is considered optimal. It should be noted that the whole column image is not shown because when the displacement is far from the focused position. Also, the contrast of the digitally refocused image decreases as in Fig. 1(b), which might cause difficulty for 3D image reconstruction. Accordingly, only the refocusing range near the focused position is considered. Nevertheless, it can be seen in Fig. 5 that some artefacts inside the reconstructed objects and surroundings remain. This might be due to the algorithm used here still being based on the contrast identification; therefore, for the case of low-contrast images, some limitations persist. Moreover, the lateral resolution of the digitally refocused image is restricted by the size of each MLA. For instance, the size of each MLA used here is 75 μm, and will shrink to around 1.875 μm on-sample after the 40X-magnification imaging system. From the experiment of the USAF targets in Fig. 1(b), group 7 can be digitally refocused and identified on-focus; hence, the lateral resolution of 1.875 μm is available for the digitally-refocused images. Nevertheless, even with this 1.875 μm resolution image, the 3D-reconstructed image for tiny and fine microstructure parts will be blurry.

### Natural Irregular and Dynamic 3D Morphology Observation

#### Micro 3D morphology reconstruction of irregular pollen grains

**Figure 6:**
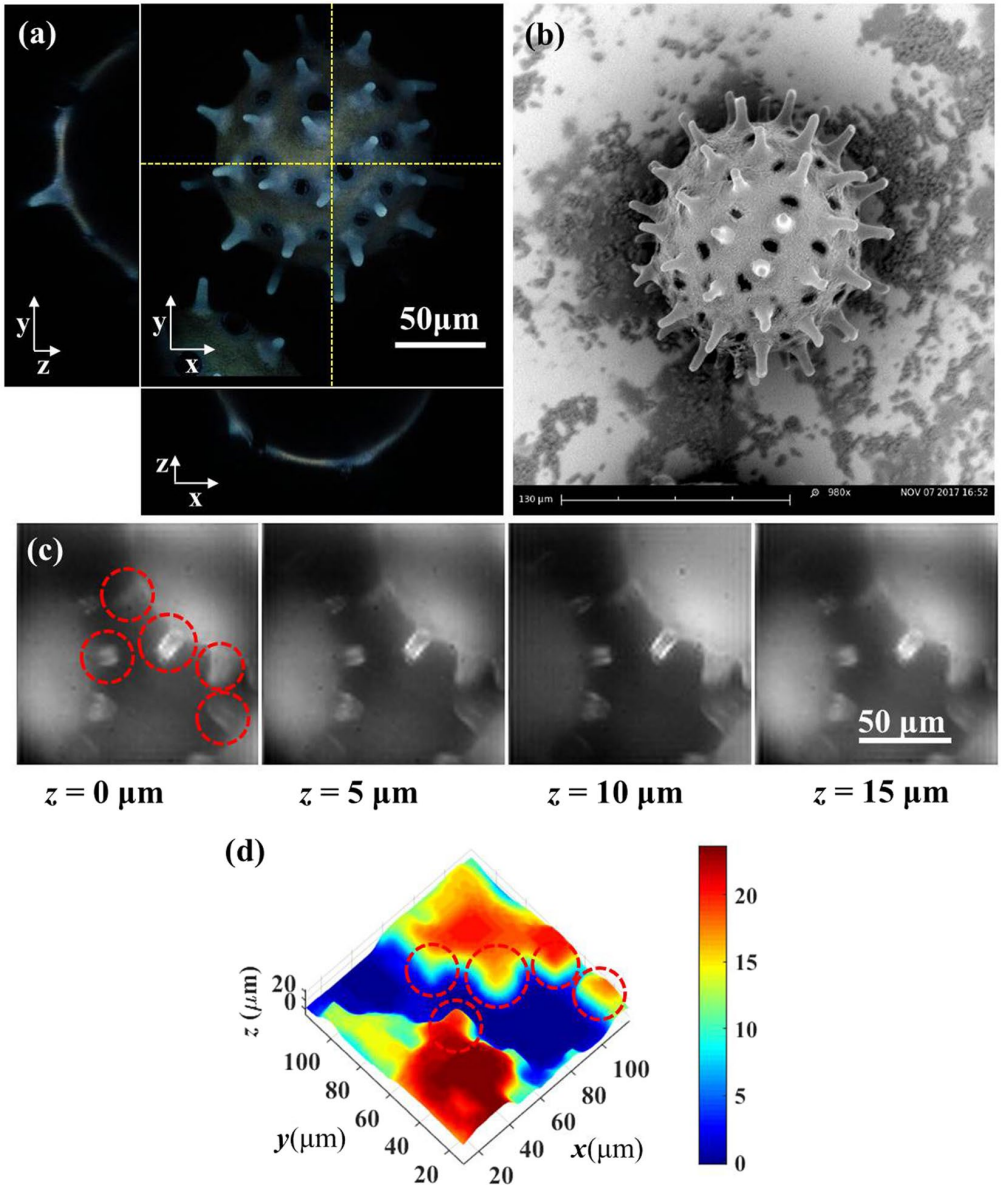
Pollen grains 3D reconstruction topography experiments. (**a**) 3D MPF image of pollen grains. 3D rendered movie is shown in Media 1. (**b**) SEM image of pollen grains. (**c**) Digital refocused image at different depth from 0 μm to 15 μm. (**d**) Isometric projection view of 3D reconstructed morphology. Rainbow pseudo color is used to illustrate different depth of object with micro-meter scale.

In this subsection, we now examine the feasibility of applying this LF microscopy and algorithm to a natural irregular object. Pollen grain was used as the target object under reflective microscopy conditions. Figure 6(a,b) are the 3D multiphoton fluorescence (MPF) image (Media 1) and the SEM image of pollen grains, respectively. The 3D MPF image is measured with the system in ref.^30^, which involves a 780 nm excitation wavelength and around 8 × 10^15^ W/cm^2^ power density on the sample. The parameters of the multiphoton image include a 200 × 200 μm^2^ lateral scan range with 512 × 512 pixels and a 1 μm axial step for 70 layers. The scale on the *xy* and *xz* planes of Fig. 6(a) was calibrated identically. In the reflective-lighting bright field LF image-capture condition, 1004 × 1002 pixels (corresponding to 133.6 × 133.8 μm^2^ field of view) and 200 ms exposure time was applied. In Fig. 6(c), the overall refocusing depth range for the pollen grain is from −15.5 μm to 0 μm for demonstration purposes. In Fig. 6(d,e) offer different points of view for the 3D reconstructed image.

From the results of Fig. 6, the lateral sizes of the pollen grains are respectively larger than 100 μm, and the spikes on the pollen grains are around 10 μm long. In comparing Fig. 6(c) with Fig. 6(a,b), the spikes on the pollen grains are clearly visible in the digitally-refocused images. Moreover, from the 3D reconstructed image in Fig. 6(d), the characteristics of the circle-marked spikes can still be reconstructed and identified. In this case, the pollen grains are not fully-opaque objects; but here, we only consider the 3D surface morphology according to the contrast quality of digitally refocused image. When comparing the final result with Fig. 6(a), it should be noted that the refocusing depth range is limited to around 20 μm; therefore, details of vertical spike image are not completely clear due to the size of each spike being larger than this scale. Accordingly, the algorithms can still function in this case since there is no need to consider the interior condition of the object.

#### Brownian motion with 30 Hz volumetric image rate

**Figure 7:**
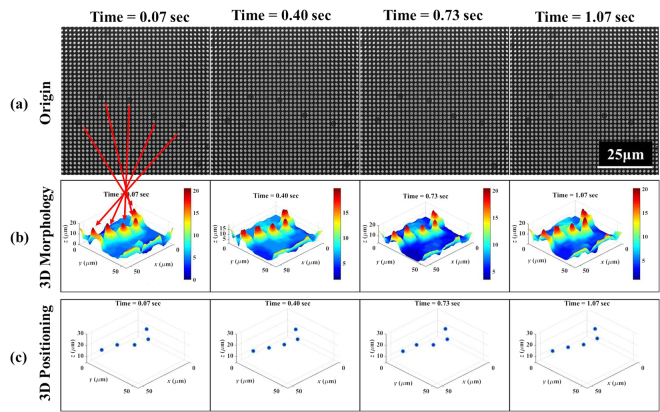
Brownian motion of 2 μm polystyrene microspheres with a 30 Hz volumetric image rate. (**a**) The original LF image at different time points; scale bar: 25 μm. The bright-field video is shown in Media 2. (**b**) The 3D reconstructed morphology at different time points. The rainbow pseudo-color is used to illustrate different depths of the polystyrene microspheres at the micron scale. (**c**) The 3D reconstructed positioning of 2 μm polystyrene microspheres at different time points. The 3D rendered video is shown in Media 3.

According to the result in Fig. 7, the high volumetric imaging rate is able to monitor the 3D Brownian motion of small particles. 2 μm polystyrene microspheres (F-8888, Thermo Fisher Scientific, USA) were used for rapid image capturing over a period of 1 second with a frame rate of 30 Hz and 512 × 512 pixels, which corresponds to a 68.3 × 68.3 μm^2^ field of view and 20 ms exposure time for reflective lighting. The overall refocusing alpha for the 3D image reconstruction ranges from 0.92 to 1, with alpha steps of 0.001, while the output image depth varies between 0 μm to 15 μm. Figure 7(a,b) respectively show the original LF image captured from the image sensor and the reconstructed 3D morphology of the polystyrene microspheres, both at different time points. The original image-sensor video is shown in Media 2. In addition, we also extend the 3D morphology into dynamic 3D positioning for observing the Brownian motion of the polystyrene microspheres. Figure 7(c) shows the 3D reconstructed positioning of 2 μm polystyrene microspheres at different time points. The 3D rendered video is shown in Media 3.

Due to only the morphology being reconstructed here, some information of the 3D image is blocked and blurred behind the polystyrene microspheres; hence, the spatial resolution in the 3D reconstruction is reduced. Moreover, the algorithm used here is suitable for a low NA condition; for a higher NA condition (larger than 0.7)^31^, the Debye theory might need to be taken into consideration. However, the behaviour of the Brownian motion can still be observed in the video, and reaches a volumetric image rate of around 30 Hz.

## Discussion

In this research, we present and demonstrate a concept for integration of different algorithm from the digital Fourier slice refocusing and contrast-dependent MRF propagation to calculate the 3D depth image. The image-quality Tenengrad-function implementation and equation (1) of an infinity optical system are used to calibrate the alpha value into practical displacement. The main concept for practical opaque 3D surface-morphology estimation in this research can be divided into different parts, as presented in the following methodology. Pre-processing includes digitally refocusing via the Fourier slice theorem with an LF image, and calibrating the alpha value with real displacements via the image quality method. The post-processing of 3D surface-morphology reconstruction includes evaluating the 3D depth image via the image contrast-dependent MRF propagation algorithm from a series of digitally refocused images, and re-mapping the depth image into a practical 3D surface-morphology image. In this work, the feasibility of applying LF microscopy and the computer vision technique was demonstrated for the 3D imaging of opaque samples. Accordingly, this proposed methodology was explored for 3D surface morphology reconstruction by imaging static artificial and natural samples, as well as dynamic 3D positioning via observing the Brownian motion of polystyrene microspheres with a 30 Hz volumetric image rate.

Based on the methodology, it was shown that practical opaque 3D image reconstruction via a one-shot LF image is feasible. Nevertheless, this method still has some limitations that cause certain artefacts and degrade the resolution of the reconstruction results. The criterion of the 3D surface morphology reconstruction is dominated by the quality of the digitally-refocused images and the adopted MRF parameters. Hence, the contrast, resolution, and signal-to-noise ratio of the digitally-refocused images might cause misjudgments in the final reconstructed morphology; in addition, the weighting parameter between the defocused and correspondence also influences the reconstruction. Regarding the optical microscope, the cutoff spatial frequencies of the defocused images decrease when far from the focal plane; hence, the spatial resolutions of the defocused digitally-refocused images worsen. Although the object on the focal plane has the best spatial resolution, it is still restricted by the size of each MLA. Moreover, the methodology used in this research is based on paraxial approximation with a low-NA (less than 0.7)^31^ and thus sacrifices the spatial resolution, but works for microstructures with coarse features. If the refocused image is too blurry, the adopted computer vision algorithm used here could misjudge the corresponding refocused plane and cause some artefacts, as shown in Figs 4 and 5. Furthermore, the computer vision algorithm is based on a window method to calculate the depth cues, which will cause ambiguities in depth measurements^26^.

In general, high resolution digitally-refocused images and the computer vision algorithm with optimal MRF parameters for judgment of the refocused plane could improve the reconstruction results. To enhance the resolution of the digitally-refocused image, an LF algorithm for a high NA-setup based on 3D deconvolution^20,23^ might be considered and adapted for non-transparent objects. With superior digitally-refocused images, more precise morphology reconstruction with fewer artefacts could be obtained. In addition, the observed field range in LF microscopy is restricted by the resolvable spot size behind each microlens^13^; hence, this limits the reconstruction range in practice. Therefore, the reconstruction range based on the current setup is around 20 μm. As such, a proper relay lens system before the MLA to minimize the resolvable spot size behind each microlens would be useful for increasing the reconstruction range^13^. With respect to the computer vision algorithm, an adaptive parameter algorithm for calculating superior MRF parameters could provide more accurate judgment of the refocused plane. Also, a non-window method may be useful to estimate the depth information.

## Methods

### Optical and Mechanical Design of Static LF Image Experiments

**Figure 8:**
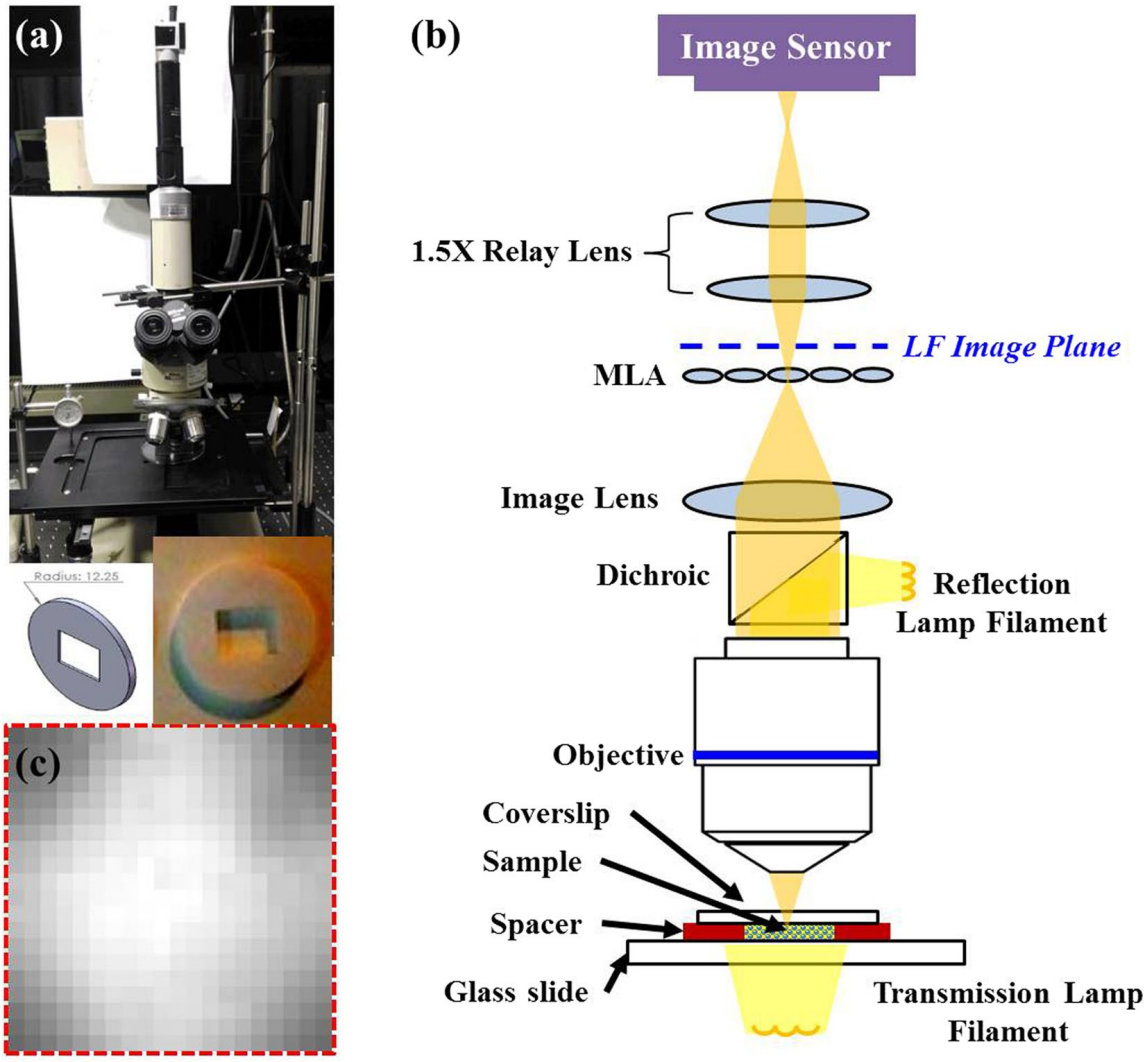
Optical configuration of LF microscopy. (**a**) System reality diagram, bottom is the design sketch and entity graph of acrylic plate with 1 mm thickness made by laser cutting, (**b**) illustration of LF microscopy. A MLA is assembled on the image plane after image lens of microscope, and then a 1:1.5 relay lens is used imaging LF image on the complementary metal-oxide semiconductor (CMOS) camera. (**c**) One image of a microlens on the CMOS sensor, around 20 × 20 pixels is covered of each microlens.

The overall infinity LF microscope design is shown as Fig. 8, and includes an objective (40X, NA = 0.65, Nikon, Japan),an upright optical microscope (Nikon, Japan), an image lens (Nikon, Japan), a C-mount spacer ring (CMSP100, ThorLabs, USA), an adapter with external SM1 threads and internal C-mount threads (SM1A10, ThorLabs, USA), and a slotted lens tube (SM1L20C, ThorLabs, USA) used to equip an MLA (Nr. 19-00020, SUSS MicroOptics SA, Switzerland). Further, a 1:1.5 matched achromatic doublet pairs (MAP105075, ThorLabs, USA) and a lens tube (SM1L15 and SM1L03, ThorLabs, USA) are used to relay the image from the back focal plane of the MLA to an image sensor, and an adapter with external C-mount threads and external SM1 threads (SM1A39, ThorLabs, USA) is used to connect the acA2040-90uc USB 3.0 camera with the CMOSIS CMV4000 CMOS sensor (acA2040-90uc, Basler, USA) via a USB 3.0 interface to a computer. A work station computer equipped with Intel Xeon ES-2630 (10 cores) was used to analyse the LF data and reconstruct 3D image. The holder utilized to mount the MLA was made by a 1 mm laser-cut acrylic plate, as shown in the bottom of Fig. 8(a).

In this optical system, shown as in Fig. 8(a,b), an MLA placed on the image plane of the microscope, separates the image into different directions on the back focal plane of the MLA. Each microlens has a 75 × 75 μm^2^ pitch and focal length is around 1 mm (in consideration to the refractive index of Fused Silica being *n*_589 nm_ = 1.458 and radius of curvature (ROC) being 0.500 mm ± 5%). To prevent direct contact of the CMOS with the MLA and applying the full pixels of images, a 1:1.5 matched relay-lens pair is used to reimage from the back focal plane image of the MLA to the one-inch CMOS sensor with a 5 × 5 μm^2^ pixel size. A reflective lighting source with a 12 V and 50 W halogen-lamp-bulb is used to offer uniform white lighting, and the exposure time of the CMOS sensor is 50 milliseconds. Finally, the result in Fig. 8(c) indicates that each MLA can capture around 20 × 20 pixels.

Herein, the high-speed automatic LF microscopy and the algorithm are used to capture images of natural pollen grains and the dynamic Brownian motion experiments. An Andor EMCCD camera (iXonEM + 885 EMCCD, Andor, UK) was used to replace the CMOS sensor for higher sensitivity with shorter exposure time, a zero electron-multiplying (EM) gain was applied, and a 40X objective (Zeiss W Plan-Apochromat 40X/1.0, Germany) was chosen in an upright optical microscope (Axio Imager.A2 m, Carl Zeiss, Germany), for which the field of view was 1004 × 1002 pixels for an area of around 133.6 × 133.8 μm^2^. A motorized stage (H101A ProScan, Prior Scientific, UK) via a 3-axis encoder and fast piezo-focusing stage (NanoScanZ 200, Prior Scientific, UK) with a maximum 200 μm traveling range is used for motorized control. All peripheral instrument communications and controls are operated via a high-speed data acquisition card (PCIe-7842R, National Instruments, USA) with Virtex-5 LX50 FPGA and a lab-made LabVIEW program.

### Integration of 4D Fourier Slicing and MRF Propagation with Defocus and Correspondence Algorithm

An LF image based on MLA can acquire information about the 2D location of a light field as well as the 2D direction. With this information, the Fourier slice algorithm in previous studies^14,32^ can be used to refocus images according to different virtual imaging planes, where the distance-ratio relations between each virtual imaging plane to image lens and MLA plane to image lens can be defined as the alpha value^14^. The MLA can also be viewed as a phase-mask modulation function, and the heterodyne LF-image refocusing method can also be applied to process refocusing. Therefore, the 4D Fourier transform is available via the 2D fast Fourier transform (FFT) and image shift^16^, after which the Gaussian resampler funciton^33^ can be used to resample the FFT of the observed sensor LF-image. The inverse Fourier transforms are processed to recover and obtain a series of virtual digitally refocused images with different alpha values.

In general, the aliasing^33^ presents an undersampling issue that results in of higher frequencies masquerading as lower frequencies in the same spectrum, and leads to obtrusive artefacts such as ghosting after refocusing^16^. Unlike the phase mask of the heterodyne LF-image, no pure spatial frequency leaks to other pure spatial frequencies^32^, and so an MLA might cause the aliasing problem. Hence, to further overcome the effects of aliasing, a post-filter of the recovered LF using a Kaiser-Bessel filter or Gaussian resampling-filter with an appropriate window width^32^ can be used, after which the optimized filter-window size and coefficient can be chosen^33^ with a different resample-filter. In this research, the Gaussian resample-filter is taken into consideration, which has good convergence speed while maintaining sufficient anti-aliasing function.

With the above series digitally refocused images, we can assume each pixel position is dependent only on different depths and independent of other pixel positions (Markovianity); moreover, due to the positive value of the image array (positivity), the low-level MRF theorem can be used to deal with this fixed grid issue. In the MRF computer vision issue, based on Markov property (Markovianity and positivity), the digital image process can be simplified to increase the feasibility of practical works. The core MRF concept is to identify a proper mathematics model that describes the probability distribution of image compositions. Traditionally, Gibbs distributions can be used to describe MRF cases. From the Gibbs framework, an energy function can be defined with respect to the MAP or ML condition^25^. To obtain the 3D depth image from the LF digitally refocused images, based on the work^26^, the refocused image can be used to calculate the defocus (*α*_D_) and correspondence (*α*_C_) images. According to the definition^26^, defocus information means the best image quality exists in the focusing, while the correspondence information reveals there is minimized variance in the focusing.

**Figure 9:**
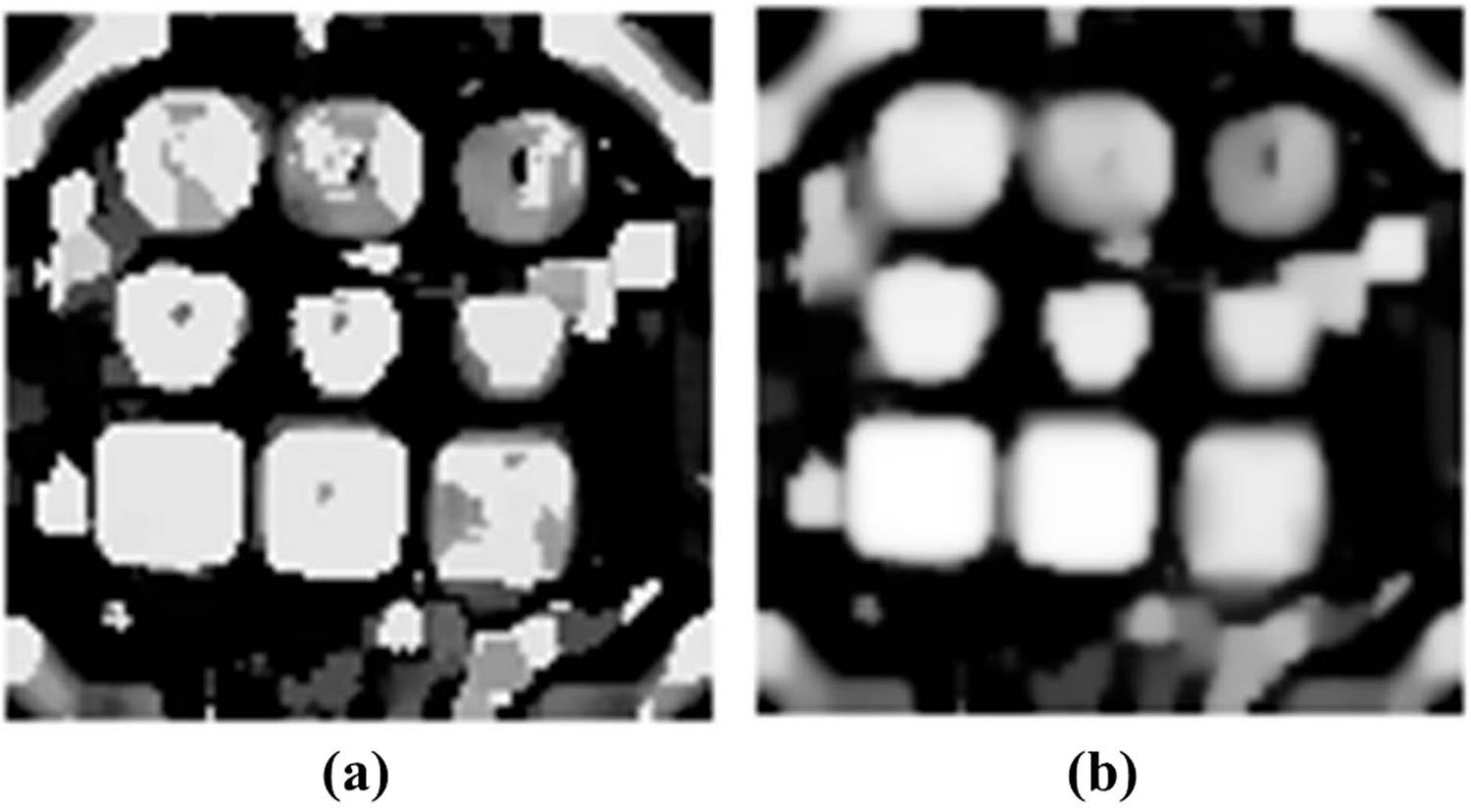
Normalized depth estimations by (**a**) only the defocus depth (*α*_D_^*^) method, and (**b**) combining the defocus depth (*α*_D_^*^) and the correspondence MRF depth (Z^*^) methods.

**Figure 10:**
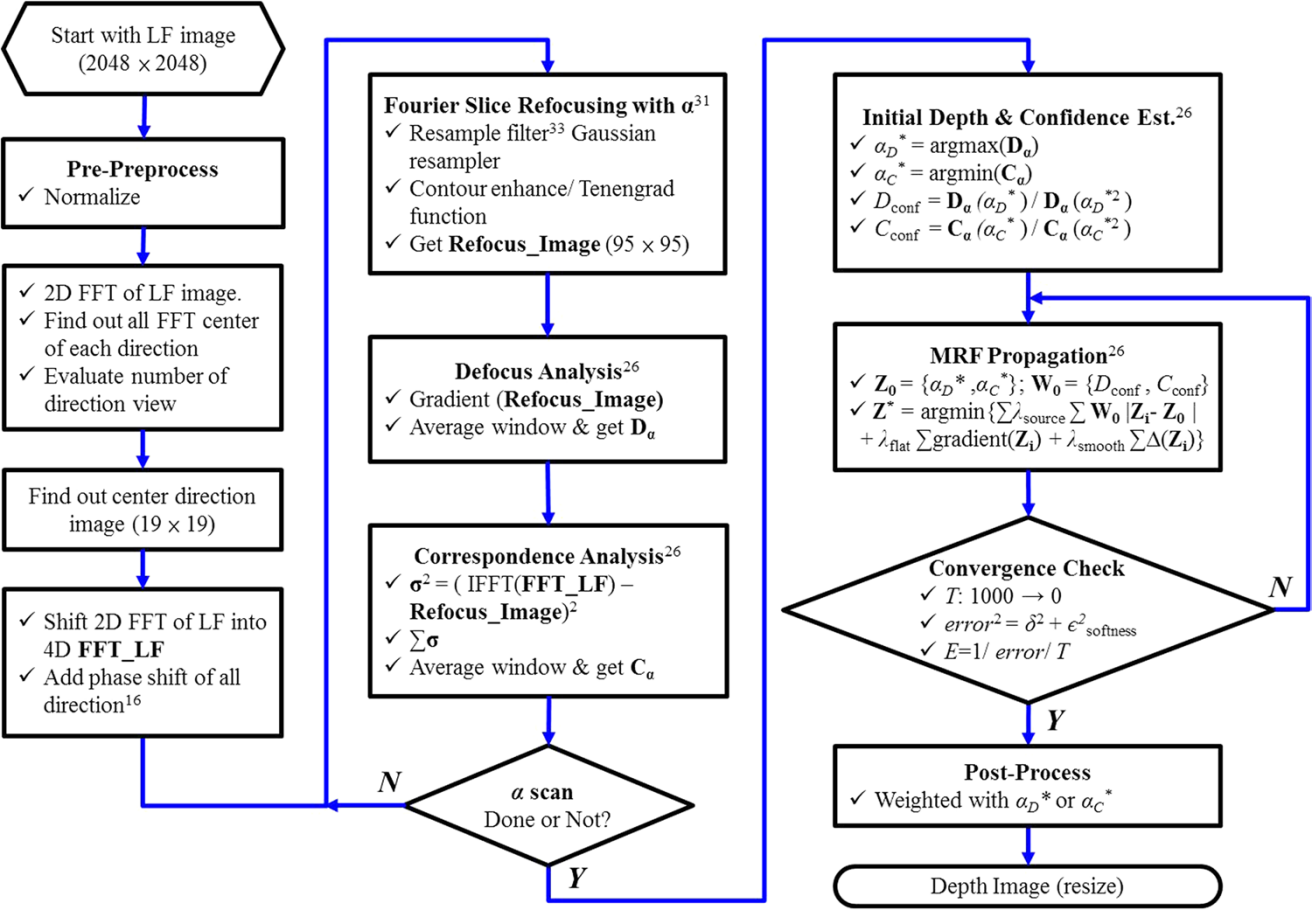
Flow-chart of integrating 4D LF Fourier slice^31^, and combining defocus and correspondence of MRF-depth estimation^26^ algorithm to calculate 3D morphology image from 2D LF-image. LF-image snapshot on image sensor is 2048 × 2048 pixels; around 19 × 19 directions is used in this analysis; Final depth image is 95 × 95 pixels. (**FFT_LF**: fast Fourier transfer of LF image array, **Refocus_Image**: digitally refocused image array, **D**_**α**_: defocused image array, **C**_**α**_: correspondence image array, *α*_D_^*^: αvalue that maximizes defocus measure, *α*_c_^*^: αvalue that minimizes correspondence measure, *α*_D_^*2^ & *α*_C_^*2^: next local optimal value or next largest peak of defocus and correspondence, *D*_conf_ & *C*_conf_: confidence value which is proportional to the ratio of response estimate α^*^ and α^*2^, **Z**_**0**_: initial array of defocus and correspondence array, **W**_**0**_: initial array of defocus and correspondence confidence array, Z^*^: depth estimation array, *λ*_source_: weighting between defocus and correspondence, *λ*_flat_: Laplacian constraint for flatness of depth estimation, *λ*_smooth_: control of second derivative kernel, *δ*: difference between Z^*^ and constraints, *є*^2^_softness_: softness of next iteration, *T*: annealing term, and *E*: error weight matrix)^26^.

Based on the work and open source codes^16,26,32^, the overall framework used to evaluate depth images in this research is shown in Fig. 9. The depth image analysis in this research can be divided into two main parts, where the first involves calculating a series of refocusing via the Fourier slice^32^ method combined with the heterodyned LF method^16^. After acquiring a series refocused images related to different virtual focusing positions, the next step is to evaluate the depth image via the defocus and correspondence method^26^. In the first part, the 2D FFT and Fourier image reshaping are used to generate a 4D Fourier LF array; then, the Gaussian resampler is used for resampling according to different alphas (*α*) to obtain a series of refocused images^32^. Each refocused image can generate one set of defocused and correspondence images^26^. After finishing all calculation for each alpha, the second part involves generation of the initial depth image from maximization of the defocus array and minimization of the correspondence array, and calculation of the confidence analysis for the initial value of the MRF propagation. Finally, the depth image calculation is the same as in ref.^26^; however, one annealing term (*T*) is added to ensure the convergence approximates to the global solution. Based on Fig. 9, two normalized depth estimations, one using only the defocus depth (*α*_D_^*^) method and the other combining the defocus (*α*_D_^*^) and the correspondence MRF depth (Z^*^) methods, are compared in Fig. 10(a,b), respectively. Obviously, Fig. 10(b) has less noise in the surrounding areas and features smoother surface morphology. Accordingly, combining these two approaches can provide a more robust method for identifying the axial focusing position^26^.

### Calculation of MRF Parameters

For MRF calculation in the case of Fig. 4, convergence parameters are softness = 1, the *λ*_source_, *λ*_flat_ and *λ*_smooth_ respectively equal 1, 2, and 2 for both defocus and correspondence, the convergence fraction = 0.5, the penalties of defocus and correspondence are respectively 0.2 and 0.8, and the window size of both defocus and correspondence is 5 × 5. In the case of Fig. 5(c,d), the convergence parameters are softness = 1, the *λ*_source_, *λ*_flat_, and *λ*_smooth_ respectively equal, 1, 1.2, and 1.2 for both defocus and correspondence, the convergence fraction = 0.5, the penalties of defocus and correspondence are respectively 0.1 and 1.2, and the window size of both defocus and correspondence is 5 × 5. Furthermore, the blue line is based on the above parameters; meanwhile, the green and red lines have the same parameters except for *λ*_flat_ and *λ*_smooth_, which are 1.1 and 1.1, and 1.5 and 1.5, respectively. In the case of Fig. 6, convergence parameters are softness = 1, the *λ*_source_, *λ*_flat_, and *λ*_smooth_ respectively equal 1, 0.5, and 0.5 for both defocus and correspondence, the convergence fraction = 0.5, the penalties of defocus and correspondence are respectively 0.6 and 0.4, and the window size of both defocus and correspondence is 5 × 5.In the Brownian motion observation which shown in Fig. 7, convergence parameters are softness = 1, the *λ*_source_, *λ*_flat_, and *λ*_smooth_ respectively equal 1, 0.5, and 0.5 for both defocus and correspondence, the convergence fraction = 0.5, the penalties of defocus and correspondence are respectively 0.8 and 0.2, and the window size of both defocus and correspondence is 5 × 5.

### 3D Surface Morphology Estimation and Calibration

**Figure 11:**
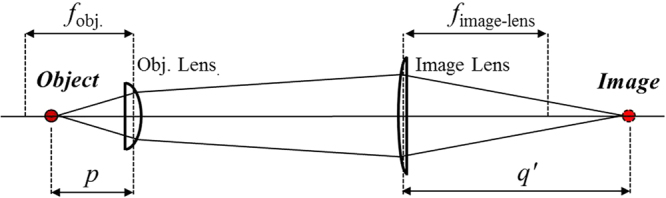
Framework of two thin lens imaging system. *f*_obj._ and *f*_image-lens_ are focal length of objective and image lens; *p* is object distance to objective; *q*′ is image distance to image lens^29^.

To calculate the practical 3D morphology image, the alpha depth image must be calibrated in advance. According to ref.^29^, the relation between the alpha and displacement can be illustrated, as shown in equation (1) and Fig. 11. Due to the infinity optical system and optics geometry, equation (1) describes the displacement of object space as ∆*p*, ∆*q*′ refers the image space displacement, *f*_obj._ is the focal length of the objective, *f*_image-lens_ is the focal length of the image lens, *M* is the magnification of the two-lens system, and ∆*α* is the difference of *α*, which is defined as the ratio of the refocused image distance to the image lens versus the distance between the focused image and image lens. The magnification *M* is usually designed as the ratio of the focal length of the two-lens pair which is defined as *M* = *f*_image-lens_/*f*_obj_.

1 $${\rm{\Delta }}p=\frac{{\rm{\Delta }}q^{\prime} }{{M}^{2}}=\frac{{f}_{{\rm{image}}-{\rm{lens}}}{\rm{\Delta }}\alpha }{{M}^{2}}=\frac{M{f}_{{\rm{obj}}{\rm{.}}}{\rm{\Delta }}\alpha }{{M}^{2}}=\frac{{f}_{{\rm{obj}}{\rm{.}}}{\rm{\Delta }}\alpha }{M}$$

From equation (1), we know the displacement relation between the object and image is linear; therefore, calibration and transferring ∆*α* into a practical distance ∆*p* is straightforward. To calibrate the relation between ∆*α* and ∆*p*, it is essential to firstly define an image quality function, which in this research is the Tenengrad function^28^. From the research^28^, the Tenengrad function has more sensitivity to contrast change or variance, and can help us precisely define the exact refocused position. The image quality evaluation versus *α* curve can be used to determine how much *α* changed versus the practical displacement of the object.

### 3D Micro Scaffold Preparation

In this research, the 3D micro-scaffolds and multiphoton image were obtained via a multiphoton fabrication system^30^. Due to its easy fine-glass attachment ability and simple manipulation, NOA-81 optical adhesive was employed to fabricate the 3D micro-structure on glass slides. After fabrication, acetone was used to remove un-solidified NOA-81 polymer; in addition, 99% ethanol and deionized (DI) water were chosen to wash the micro-scaffolds, after which they were dried at room temperature. Approximately 50 nm-thickness Ni thin film was sputtered onto the micro-structure to increase the reflectivity of the scattering light. If needed, a multiphoton scanning system was used to re-process the Ni-coated 3D micro-scaffolds to remove any background Ni on the glass slide via optical etching to increase the contrast of the image. The 3D scaffolds were designed via the commercial software SolidWorks, and pre-sliced for fabrication via the “Matlab STL GUI slice program” (developed by Y. D. Sie - source: Matlab-Central).

### 3D Reconstructed Positioning of Brownian Motion

The Brownian motion of 2 μm polystyrene microspheres from the original 3D morphology reconstruction can be tracked with a 30 Hz volumetric image rate. The 3D reconstructed positioning steps are: (1) choose an appropriate thresholding in Fig. 7(b) to eliminate as much background noise as possible; (2) find the local-maximum peak location in the thresholding image of Fig. 7(b); and, (3) extract the 3D position coordinates as the 3D spatial distribution of the polystyrene microspheres.

## Electronic supplementary material

Video 1

Video 2

Video 3
